# Minireview of Epilepsy Detection Techniques Based on Electroencephalogram Signals

**DOI:** 10.3389/fnsys.2021.685387

**Published:** 2021-05-20

**Authors:** Guangda Liu, Ruolan Xiao, Lanyu Xu, Jing Cai

**Affiliations:** College of Instrumentation and Electrical Engineering, Jilin University, Changchun, China

**Keywords:** epilepsy, neurological disorder, EEG, machine learning, detection

## Abstract

Epilepsy is one of the most common neurological disorders typically characterized by recurrent and uncontrollable seizures, which seriously affects the quality of life of epilepsy patients. The effective tool utilized in the clinical diagnosis of epilepsy is the Electroencephalogram (EEG). The emergence of machine learning promotes the development of automated epilepsy detection techniques. New algorithms are continuously introduced to shorten the detection time and improve classification accuracy. This minireview summarized the latest research of epilepsy detection techniques that focused on acquiring, preprocessing, feature extraction, and classification of epileptic EEG signals. The application of seizure prediction and localization based on EEG signals in the diagnosis of epilepsy was also introduced. And then, the future development trend of epilepsy detection technology has prospected at the end of the article.

## Introduction

Epilepsy is a neurological disorder caused by the sudden abnormal discharge of brain neurons. The typical characteristics of epilepsy are recurrent, unconscious body movements, and so on (Sra et al., 2019). Uncontrollable seizures are more likely to induce Depression, Cardiovascular disease, and other diseases making patients and their families miserable (Supriya et al., 2020). The World Health Organization (WHO) report manifests that approximately 50 million people have epilepsy worldwide (Liu et al., 2020). Knowing the precursors of epilepsy can allow patients to avoid the pain of epileptic seizures through drug control, so there are an urgent need for simple, fast and effective epilepsy detection methods.

EEG is a commonly used non-invasive auxiliary method in the clinical diagnosis of epilepsy. However, it is a highly tedious, laborious, time-consuming, and costly task for neurologists to identify seizures from EEG for a long time (Yao et al., 2021). Therefore, it is necessary to develop a reliable epilepsy automatic detection system, which can significantly improve the quality of life of epilepsy patients (Solaija et al., 2018). Prior et al. (1973) introduced the Cerebral Function Monitor (CFM) that monitors the long-time EEG, which can record the number of seizures. Subsequently, Gotman (1982) selectively recorded the EEG signals of epileptic in the interictal and ictal as samples and used its amplitude, period, and other characteristics to distinguish whether the samples were in the state of epileptic seizures. Martinerie et al. (1998) realized the epileptic seizure detection by extracting the non-linear indicators of the EEG signal around seizure onset. The above work uses computers to automatically collect EEG data of patients with epilepsy and try to extract the characteristics of the lesions, but it cannot fully realize the prediction of epileptic seizures, and it is of limited help to medical staff and patients. While the emergence of machine learning promotes the development of epilepsy detection techniques (Aayesha et al., 2021), it provides the possibility to automatically detect epilepsy, thus attracting more and more researchers to join it. Chen et al. (2014) proposed an epilepsy detection framework based on machine learning to realize epileptic seizure detection. Later Craley et al. (2021) developed an end-to-end deep learning model for automatic seizure detection in multichannel EEG recording. Their outstanding research has made machine learning a step toward success in the field of automatic epilepsy detection. At present, most patients with epilepsy can be treated with drugs (Wang et al., 2021). Liu et al. (2018) and Ito et al. (2021) used EEG observing epileptic discharges to verify anti-epileptic drugs’ reliability. For patients with drug-resistant epilepsy, surgical treatment, such as Temporal Lobectomy (TL) is necessary to control seizures (Anoop et al., 2021). And for patients with refractory epilepsy, Vagus nerve stimulation (VNS) has a significant therapeutic effect (Shimogawa et al., 2021). Under such circumstances, using machine learning algorithms in EEG signals to realize epileptic detection, thus realizing the treatment effect evaluation, will help clinicians treat epileptic (Assi et al., 2017; Al-Hadeethi et al., 2020).

**FIGURE 1:**
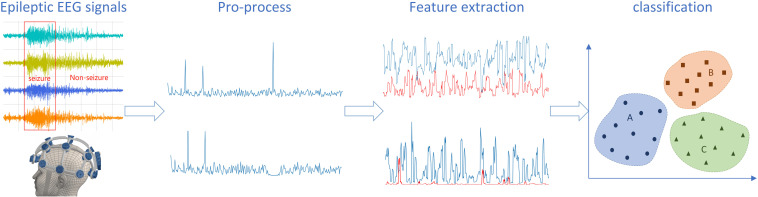
The key steps of epilepsy detection.

The machine learning algorithm mainly compares the abnormal time-frequency domain characteristics of the EEG signal of patients with epileptic seizures to detect epileptic seizures. In recent years, seizure detection has also promoted the development of seizure prediction and location. This minireview introduced the decisive steps of epileptic seizure detection shown in Figure 1, including the acquisition, preprocessing, feature extraction, and classification of epilepsy EEG signals, besides the application of seizure prediction and localization in the diagnosis of epilepsy and trend of future seizure detection techniques was also given here.

## Acquisition of EEG and Preprocessing

There are many devices for obtaining EEG signals, such as brain-computer interface (BCI) equipment from Neurosky and portable EEG acquisition equipment by COMPUMEDICS NeuroScan. The EEG signals can be acquired by placing EEG electrodes on the scalp of patients with epilepsy. EEG electrodes can be placed on the whole brain according to the international 10–20 EEG system for EEG electrode placement (Herwig et al., 2003).

Before detecting the collected epilepsy EEG signals, it is a regulation method to use the publicly available epilepsy EEG signal data set (e.g., epilepsy EEG dataset of Children Hospital Boston, Massachusetts Institute of Technology (CHB-MIT), epilepsy EEG dataset of The Freiburg, epilepsy EEG dataset of Bonn University) to establish and verify the EEG detection model. In addition to using publicly available datasets, some researchers used clinical epilepsy EEG data by clinicians to verify the reliability of the epilepsy detection model (Iesmantas and Alzbutas, 2020). Furthermore, some researchers evaluated the model’s reliability through cross-database (Sun et al., 2019). Raghu et al. (2020) used five different epilepsy EEG datasets for the first time to verify the generalization capability of seizure detection models. What’s more, the collected epilepsy data needs to undergo preprocessing, including artifacts removing and noise filtering, to obtain a clean epilepsy EEG signal for the next step, feature extraction (Acharya et al., 2018a).

## Feature Extraction

Feature extraction is an essential step in epileptic seizure detection, which is used to establish an epilepsy detection model via standard epilepsy data, and epilepsy detection from actual collected EEG signal data. The effect of feature extraction is closely related to the accuracy of epilepsy detection, so it is imperative to improve feature extraction. And research shows that different dimensionality reduction methods can improve the saliency of features. Al-Hadeethi et al. (2020) proposed for the first time to use the covariance matrix for reducing EEG signals dimensionality and extract its statistical features, and use non-parametric tests to obtain the set that has the most distinguishing features, which can be used as the input of Adaptive Boosting Least Square-Support Vector Machines (AB-LS-SVM) model to achieve satisfactory results (>99% accuracy). Vicnesh and Hagiwara (2019) extracted the non-linear features from the EEG data, selected them, and then fed them into the decision tree (DT) to classify the different epilepsy classes.

EEG signals are non-linear and non-stationary time signals. Using wavelet transform to re-express EEG signals is a commonly used method of dimensionality reduction. Ott et al. (2010) extracted the standard deviation, variance, and higher-order moments after wavelet transform and used them as the input of linear discriminant analysis (LDA) and k-nearest neighbor (KNN) classifiers. On CHB-MIT, the method yielded a classification accuracy of 99.45% using the KNN classifier. Wang et al. (2017) presented a three-class classification system based on discrete wavelet transform (DWT) and the non-linear sparse extreme learning machine (SELM). Three-level lifting DWT using Daubechies order four wavelets was introduced to decompose the Bonn University EEG dataset, and the maximum and standard deviation values of each subband were computed. The experiment obtained a classification accuracy of 98.4%. DWT was also used by Amin et al. (2020) for differentiating epileptic seizures for standard signals.

In addition to wavelet transform, the transformation of epilepsy EEG signals can also use empirical mode decomposition (EMD), wavelet packet decomposition (WPD), etc. A new method was presented by Ahmet and Aydin (2018) to analyze intrinsic mode functions (IMFs) decomposed by EMD. The method showed 97.89% accuracy by using the Bonn University EEG dataset. Ailckovic et al. (2018) applied EMD, DWT, WPD to process the Freiburg and CHB-MIT EEG dataset and extracted six statistical features, after that putting them into Random Forest (RF), Support vector machine (SVM), Multilayer perceptron (MLP), and KNN classifiers. This method could discriminate between inter-ictal and pre-ictal EEG states with an accuracy of 99.70%. Hassan and Subasi (2016) proposed a new signal processing scheme for EEG signal segments, namely complete ensemble empirical mode decomposition with adaptive noise (CEEMDAN). Six spectral moments were extracted from the CEEMDAN mode functions and then inputted into linear programming boosting (LPBoost) classifier. This model got 100% accuracy. Tao et al. (2017) applied a fusion method of variational mode decomposition (VMD) and autoregression (AR) for feature extraction. Statistical features of the best AR model coefficients were calculated and fed into RF classifier for classification. Finally, the test showed 97.352% accuracy. Another critical step after feature extraction is classification, which will give the final epilepsy detection results from the extracted features.

## Classification

In recent years, the application of machine learning in the classification of epilepsy diagnosis has attracted more and more researchers. And some machine learning algorithms used in epilepsy detection and classification are summarized in Table1, mainly including SVM, convolutional neural networks (CNN), extreme learning machines (ELM), and other algorithms.

**TABLE 1:** Summary of machine learning methods for epilepsy detection.

**Author**	**Dataset**	**Model**	**Accuracy (%)**
Janjarasjitt and Suparerk	CHB-MIT	SVM	96.87
Chen et al.	BONN	LS-SVM	99.5
Al-Hadeethi et al.	BONN	AB-LS-SVM	99
Qi et al.	BONN	ELM	96.5
Li et al.	BONN	M-ELM	100
Song et al.	BONN	FF-ELM-SD	97.53
Wang et al.	BONN	SELM	97.6
Acharya et al.	BONN	CNN	88.67
Wei et al.	CHB-MIT	CNN	90.57
Nogay and Adeli	CHB-MIT	DRNN	100
Choubey and Pandey	BONN	ANN + KNN	KNN:98 ANN:94
Yuan et al.	CHB-MIT	BLDA	95.74
Zeng et al.	BONN	GRP-DNet	100
Juarez-Guerra et al.	BONN	MRW-FFWNN	95.0

SVM is a commonly used classifier, and the classification results of which can be changed using different kernel functions and different cross-validation multiples. Janjarasjitt (2017) applied SVM to classify single-channel scalp EEG data features times. Moreover, the test got an average classification accuracy rate of 96.87% using 10-fold cross-validation. Besides, many researchers combine different algorithms with SVM to obtain better classification accuracy and detection efficiency. Makaram et al. (2020) extracted the time domain characteristics and signal complexity. Further, they used the Support Vector Machine-Error-Correcting Output Codes (SVM-ECOC) to train the classification algorithm, and the improvement in classification accuracy had been obtained. Ramakrishnan and Murugavel (2019) proposed a new seizure detection model using layered directed acyclic graph SVM (LDAG-SVM), which improved classification accuracy and reduced detection time compared to existing methods. After performing DWT, Chen et al. (2019) extracted the non-linear features of each sub-band and inputted them into six different classifiers for training. Finally, they increased the classification accuracy of Least Square-Support Vector Machines (LS-SVM) to 99.5%, which was better than five other classifiers. Based on LS-SVM, Al-Hadeethi et al. (2020) further applied the AB-LS-SVM model for epilepsy detection.

In 2006, Huang et al. (2006) improved Backward Propagation (BP) to improve learning efficiency, simplify learning parameters, and proposed ELM. Then Qi et al. (2011) extracted non-linear features and applied ELM for an epilepsy diagnosis. The classification accuracy was improved to 96.5%, which was better than BP and SVM in both classification accuracy and training time. To make ELM better application, Li et al. (2016) proposed a ternary classification system based on the Multiplicative Extreme Learning Machine (M-ELM), with a maximum classification accuracy of 100%. Song et al. (2016) designed a novel fusion feature and integrated the fusion feature and ELM. Experimental results demonstrated 97.35% classification accuracy. Wang et al. (2017) applied SELM for epilepsy detection. Liu et al. (2017) proposed Kernel ELM and introduced Cholesky decomposition to reduce the computation of out weights. The experimental results showed that the method can achieve an average classification accuracy of 96.5%. On this basis, Zhang et al. (2019) proposed Expectation Kernel ELM (EKELM) to further improve ELM classification abilities.

In 2018, Acharya et al. (2018b) applied CNN to the study of EEG signals for the first time and realized a 13-layer deep convolutional neural network for epilepsy detection without separate feature extraction and feature selection. The proposed technique achieved an accuracy of 88.67%. Iesmantas and Alzbutas (2020) extracted different features from clinical epilepsy EEG signals and applied CNN for training data. Wei et al. (2019) used the increasing and decreasing sequences (MIDS) merger to highlight the characteristic of waveforms and a data augmentation method for increasing the sample diversity and EEG information. Furthermore, they applied CNN classifier for epilepsy detection to get 90.57% accuracy. Nogay and Adeli (2020) proposed a machine learning method for seizure detection using the pre-trained deep two-dimensional CNN and transfer learning concept that achieved 100% accuracy for binary classification and ternary classification for epileptic seizure detection.

With the continuous development of machine learning, new algorithms are constantly being introduced into seizure detection. Akyol (2020) proposed a new deep neural network for seizure detection that successfully obtained an average accuracy of 97.17%. Choubey and Pandey (2020) used Artificial Neural Network (ANN) and KNN to achieve seizure detection. Yuan et al. (2018) applied a Bayesian linear discriminant analysis (BLDA) classifier to classify the CHB-MIT scalp EEG dataset and achieved an average classification accuracy of 95.74%. Zeng et al. (2021) combined gray recurrence plot (GRP) and densely connected convolutional network (DenseNet) for epilepsy detection and even achieved 100% excellent classification accuracy in each classification experiment. Mouleeshuwarapprabu and Kasthuri (2020) proposed a Non-linear Vector Decomposed Neural Network (NVDN) detect epileptic seizures and obtained 95.60% effective epilepsy detection results. Sharma et al. (2020) described a computationally fast seizure classification algorithm using non-linear higher-order statistics and deep neural network algorithms. This technique could capture weak information related to epilepsy EEG signals and achieved 100% seizure classification accuracy. Juarez-Guerra et al. (2020) proposed a new epilepsy seizure detection method for classifying epilepsy seizures, namely Multidimensional Radial Wavelons Feed-Forward Wavelet Neural Network (MRW-FFWNN). The experiment showed that the accuracy of the three classifications was 93.33%. From the above research results, it is not difficult to find that the research on epilepsy detection has been fruitful, and even some epilepsy detection algorithms have reached 100% accuracy. However, scientists’ research on epilepsy does not stop there. The goal they really want to achieve is to prevent it before it happens, in other words, to predict epilepsy.

## Seizure Prediction and Localization

In the 1970s, seizure prediction has become a hot research topic (Assi et al., 2017). MohanBabu et al. (2020) focused on the seizure prediction obtained from the CHB-MIT scalp EEG dataset using an optimized deep learning network model (ODLN), and the experiment by them provided 100% accuracy of seizure prediction. Zhang and Parhi (2016) extracted 44 features every 2 s for each channel and then ranked and selected them in a specific way. The selected features were processed by the Kalman filter and then inputted into the SVM classifier. This algorithm could achieve 100% sensitivity on the Freiburg EEG dataset. Daoud and Bayoumi (2019) applied deep learning to achieve epileptic seizure prediction, achieving epileptic seizure prediction while attaining 99.9% accuracy of epileptic seizure prediction.

Identifying epileptogenic zones prior to surgery is an indispensable step for patient before surgery. Alshebeili et al. (2020) proposed a framework that uses DWT and SVM to solve the problem of focus positioning. The framework used the best frequency band characteristics and wavelet coefficient characteristics, and its positioning accuracy could reach 88.0%. Sriraam and Raghu (2017) extracted 26 features from focal and non-focal EEG, then they used Wilcoxon rank sum test to select significant features and used an optimized SVM classifier with 10-fold cross-validation to perform important functions classification. This method achieved an accuracy of 92.15% and could be used to identify focal EEG signals to locate epileptic areas. Myers et al. (2020) also proposed a novel method for automatic localization of seizure on the scalp from clinical EEG data, which could get 93.3% accuracy and 100% sensitivity. Wu et al. (2021) presented a new localization method for epileptic seizure onset zones (SOZs), an unsupervised clustering method based on the combination of adaptive-genetic-algorithm-based matching pursuit (AGA-MP) and k-medoids clustering method. Moreover, compared with several existing methods, this method had certain advantages in sensitivity and specificity.

## Other Applications of Machine Learning

Machine learning algorithms applied to EEG signals have also shined in other fields. Seal et al. (2021) used deep CNN to detect Depression and the detection accuracy of this algorithm was 99.37%. While Zhang et al. (2021) used CNN for motor imagery (MI) classification, and the average accuracy of the model reached over 88.4%. Huang (2021) recognized different psychological emotions via improved SVM, whose classification accuracy was as high as 85.9%. Raurale et al. (2021) develop an automated system combining quadratic time-frequency distribution (TFD) with CNN to identifying the severity of hypoxic-ischemic encephalopathy injury (HIE), which could assist clinical decision-making for neonates with HIE.

## Conclusion

Since the beginning of the twenty-first century, the rapid development of artificial intelligence and machine learning, epilepsy detection techniques based on EEG signals has attracted more and more attention from researchers. This minireview briefly introduced the basic idea of epilepsy detection techniques based on EEG signals. From epilepsy EEG data and preprocessing to feature extraction and classification, the research progress of epilepsy automatic detection techniques in recent years were reviewed. Due to the random nature of epileptic seizures, fast and convenient seizure detection is essential for the immediate treatment of epilepsy patients. There is still much room for the development of epilepsy detection techniques. Here are a few points about the future development trend of epilepsy detection techniques based on EEG signals.

1. Seizure prediction and localization are still one of the future development directions of epilepsy detection techniques. Seizure prediction can effectively improve the quality of epilepsy patients, and non-invasive epilepsy focus localization can better assist clinicians in epilepsy diagnosis time and save costs.
2. Epilepsy detection is related to the patient’s age, region, and other things, but the publicly available epilepsy EEG datasets are limited. Therefore, many epilepsy clinical EEG data from different countries and different countries and different age groups need to be improved.
3. With the development of machine learning, more and more new methods are applied to the feature extraction and classification of epilepsy EEG signals. The emergence of deep learning may gradually replace machine learning as the mainstream epilepsy diagnosis method in the future.
4. With wireless transmission technology development, seizure detection may get rid of wired transmission in the future and realize remote epilepsy detection.

In recent years, more and more new methods have begun to be applied to the automatic detection of epilepsy. The development of faster and more accurate epilepsy detection models will contribute to epilepsy detection techniques in clinical diagnosis and the development of portable and integrated epilepsy detection equipment. Therefore, a concise and efficient epilepsy detection model will become an inevitable development trend in the future.

## Author Contributions

All authors listed have made a substantial, direct and intellectual contribution to the work, and approved it for publication.

## Conflict of Interest

The authors declare that the research was conducted in the absence of any commercial or financial relationships that could be construed as a potential conflict of interest.
